# Enhanced Hydrogen-Rich Syngas Production Through In-Situ Heavy Oil Gasification Process Using Nanoscale Nickel Catalyst

**DOI:** 10.3390/molecules30040809

**Published:** 2025-02-10

**Authors:** Tiantian Wang, Renbao Zhao, Ying Yang, Haitao Ren, Wentao Lv, Han Xu, Jiyang Liu

**Affiliations:** 1 State Key Laboratory of Petroleum Resources and Engineering, China University of Petroleum, Beijing 102249, China; wttcup21@163.com (T.W.); 15171123882@163.com (Y.Y.); 15935903697@163.com (H.R.); 2022215215@student.cup.edu.cn (W.L.); 13396916979@163.com (H.X.); 17550229017@163.com (J.L.); 2 MOE Key Laboratory of Petroleum Engineering, China University of Petroleum, Beijing 102249, China

**Keywords:** hydrogen energy, hydrogen-rich production, heavy oil, nickel catalyst, in-situ combustion gasification process

## Abstract

With the increasing demand for clean energy, in-situ hydrogen production from hydrocarbon reservoirs has attracted increasing attention. In this work, a nanoscale nickel catalyst was prepared using the water-in-oil (w/o) microemulsion method and applied in the in-situ generation of hydrogen-rich syngas from heavy oil reservoirs. The activation energy (Ea) of the gasification reactions significantly decreased with the addition of the nickel catalyst. The catalytic effect was monitored through remarkable increases in the peak temperature values for both the low-temperature oxidation (LTO) and high-temperature oxidation (HTO) processes, and the two peaks also shifted to lower-temperature regions. Additionally, the catalyst exhibited excellent activity and selectivity during the reaction process, and therefore the highest production rate of hydrogen-rich syngas of 20.07%, combined with the peak hydrogen concentration of 5.00%, was obtained in the presence of the catalyst and water. The substantial rate of hydrogen conversion from heavy oil was calculated to be 397.87 mL/g. The preliminary results obtained in this work show that this method is a significant improvement, and the catalyst-assisted method is believed to have great potential for underground fossil fuel conversion in the future.

## 1. Introduction

With the global advocacy for carbon reduction, hydrogen energy is quickly becoming a prominent clean energy source. The conversion of fossil fuels, such as heavy oil, into hydrogen-rich syngas (H_2_, CH_4_, and CO) and CO_2_ is considered an attractive method for hydrogen generation, as hydrogen is normally a major component of the emission gas mixture [1,2]. In-situ hydrogen generation from petroleum reservoirs is believed to be a significant solution for achieving both the decarbonization of the petroleum industry and clean hydrogen production [3]. Using this promising approach, hydrogen-rich syngas is extracted while all the carbon products are sequestrated underground through the down-hole separation process [4]; hence, the surface environmental impact is significantly minimized or avoided due to this in-situ sequestration. Holladay et al. [5] reported that in-situ hydrogen or hydrogen-containing gas mixtures can be generated in heavy oil reservoirs via thermal cracking, aqua thermolysis, water–gas shifts, and coke gasification during air injection. Kapadia et al. [6] developed a new kinetic model to examine the potential for hydrogen generation from Athabasca bitumen. Tang et al. [7] showed that metal oxides have catalytic effects on the in-situ generation of hydrogen from heavy oil during the gasification process. However, current hydrogen production still has a relatively low concentration; therefore, further research is required to enhance the reactivity of various hydrogen production reactions to achieve a higher concentration.

In order to obtain a higher hydrogen concentration and a higher hydrogen conversion efficiency during the heavy oil gasification reaction, a catalyst is normally injected into the oil reservoir. Recently, nickel-based catalysts have been explored to optimize hydrogen production from heavy oil reservoirs, as they can shift the hydrogen generation reaction to a lower temperature and therefore significantly reduce energy consumption [8,9,10]. Tomita et al. [11] reported that the hexamine nickel (II) carbonate catalyst can reduce the gasification temperature to around 480 °C, with hydrogen being the main gas product. Furthermore, nickel catalysts are believed to lower the activation energy required for heavy oil cracking by promoting the breakage of C-S, C-N, and C-C bonds, as well as some C-H bonds, which is a key step in the catalytic hydrogen production reaction. Yi et al. [12] found that the addition of a nickel nitride (NiN) catalyst increases the H_2_ concentration from 0% to 1.5%, while the CO concentration ranges from 0% to 1.2% and is accompanied by an increase in CH_4_, from 39.5% to 40.4%, during the hydrothermal pyrolysis of asphaltenes and resins. Rana et al. [13] showed that 4.46 mol/kg of hydrogen is produced from light oil, and this is combined with a total gas generation efficiency of 9.22 mol/kg. However, there are various challenges in the application of nickel-based catalysts. Nickel oxide (NiO), for example, is commonly employed as a precursor of nickel catalysts; however, its catalytic activity cannot be activated until the reduction process from Ni^2+^ to Ni^0^ is initiated. Nickel nitride (NiNx) shows reduced activity at higher ambient temperatures and is prone to decomposition. Nickel sulfide (NiSx) is particularly sensitive to sulfur loss, which leads to decreased catalytic activity and unstable performance. A nickel catalyst with a higher percentage of nickel atoms achieves significantly enhanced catalytic performance by providing a greater density of active catalytic sites [14]; therefore, further research is crucial to fully explore its capabilities, especially in the catalysis of heavy oil cracking through an in-situ gasification process for hydrogen-rich syngas production.

Despite the excellent catalytic performance of nickel catalysts, deactivation induced by high-temperature sintering, in which catalyst particles are prone to aggregation (of even larger particles) under high-temperature conditions, is regarded as a significant challenge. The active surface area is noticeably reduced, and there is a significant decrease in catalytic activity [15]. Nanoscale catalysts, which effectively mitigate sintering under high-temperature conditions, are synthesized using various methods, such as microemulsion, precipitation, sol–gel processing, chemical vapor deposition, and electrochemical deposition [16,17,18]. Among these, reverse microemulsion (also known as the water-in-oil (w/o) type) is regarded as an isotropic and thermodynamically stable colloidal system and is widely used in the preparation of nanoparticles [19,20]. In a given reverse microemulsion system, nanoscale water droplets are dispersed in a continuous oil phase and can serve as nanoscale reactors for the preparation of nanocatalysts. A surfactant solution with a concentration higher than that of the critical micellar concentration (CMC) is likely to form aggregated structures known as micelles [21]. Water droplets can then be solubilized in the micelles to form swelling micellar structures, and the nanoscale particles are dispersed in the continuous oil phase. In addition, the surfactant can also inhibit the agglomeration of liquid particles, thereby forming a nanoscale dispersed colloidal system with a thermodynamically stable state [22]. Therefore, a reverse microemulsion colloidal system offers a promising approach for synthesizing nanoscale catalysts and mitigating their deactivation in high-temperature environments.

In this work, the synthesis of nanoscale nickel catalysts and their catalytic performance in hydrogen-rich syngas production during an in-situ gasification process were studied. The catalytic activity, thermal stability, and hydrogen production selectivity of the nanoscale nickel catalyst were systematically investigated by analyzing activation energy fingerprints, reaction kinetics, and hydrogen-rich gas production efficiency. The results demonstrate that the nanoscale nickel catalyst was proven to be an attractive material for promoting the generation of hydrogen-rich syngas during the gasification of heavy oil.

## 2. Results and Discussion

### 2.1. Characterization of the Catalyst

**Figure 1:**
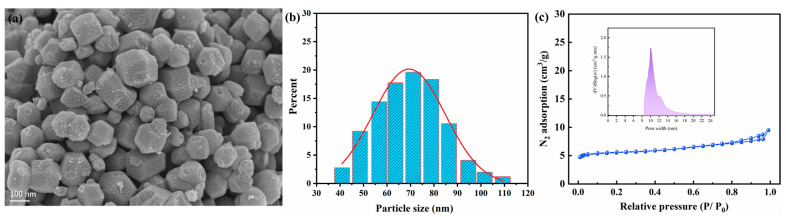
Characterization of the nanoscale nickel catalyst: (**a**) an SEM micrograph of the catalyst’s surface, (**b**) the particle size distribution derived from SEM image analysis, and (**c**) the N_2_ adsorption isotherm and pore size distribution.

The surface morphology and particle size distribution of the prepared catalyst were characterized by SEM, as shown in Figure 1. The SEM image indicates a cubic morphology for the prepared catalyst, which is consistent with reports in the literature [23,24]. The particle size distribution of the nickel catalyst indicates the average particle size falls into the range of 50–90 nm. The N_2_ adsorption/desorption isotherm and particle-assembling pore size distribution of the nano-nickel are shown in Figure 1c. The sample exhibited a type IV isotherm. The specific surface area of nano-nickel is 8.6 m^2^/g. This relatively high specific surface area is mainly attributed to the small size distribution of the catalyst particles, which increases the area in contact with heavy oil and enhances the catalytic activity [25].

### 2.2. Catalytic Activity Evaluation

**Figure 2:**
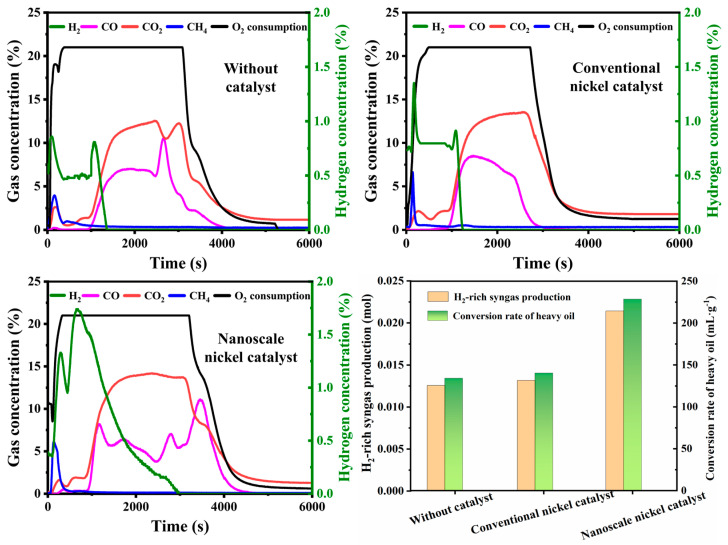
The effluent gas compositions resulting from in-situ gasification of heavy oil with no catalyst, a conventional nickel catalyst, and a nano-nickel catalyst.

The reaction characteristics of hydrogen-rich syngas production during the in-situ gasification of heavy oil with conventional and nano-nickel catalysts, as well as without a catalyst, were compared. The concentrations of the effluent gases were continuously measured online and are plotted in Figure 2. In the initial stages of the experiments, the O_2_ consumption significantly increased while the gases CO, CO_2_, and CH_4_ rose gradually. Then, the CO_x_ concentration rapidly increased to a higher level at 1500 s, oscillated in the range of 5–12%, then gradually decreased, as the O_2_ consumption showed a decreasing trend in the final stages of the experiments. The maximum H_2_ concentrations for the experiments with no catalyst, a conventional nickel catalyst, and a nano-nickel catalyst were measured to be 0.86%, 1.35%, and 1.74%, respectively. The durations of the hydrogen generation process for the three cases were approximately 1700 s, 1700 s, and 3000 s, respectively. Furthermore, the H_2_-rich gas production and the hydrogen conversion rate of heavy oil are also presented in Figure 4. The results show that the highest H_2_-rich gas production of 0.021 mol, combined with a hydrogen conversion rate of 228.54 mL/g, was achieved in the experiment with the nano-nickel catalyst. The excellent performance of nano-nickel catalysts is attributed to their more active catalytic sites, which greatly increase the contact area between heavy oil and nanoparticles.

**Figure 3:**
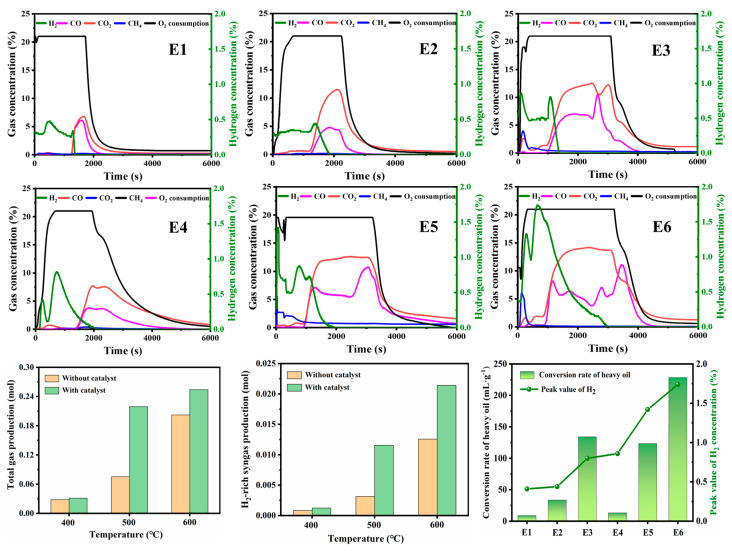
Variation in effluent gas composition over time in different experiments.

The performance of the nanoscale nickel catalyst for hydrogen-rich syngas production varied in the temperature range of 400–600 °C, as depicted in Figure 3. The CH_4_ concentrations for experiments E1 and E2 were approximately 0.32% and 0.45%, respectively, and both oscillated to a small degree. However, this concentration significantly increased to about 3.96% in experiment E3, which was attributed to the thermal cracking of heavy oil at an ambient temperature of 600 °C. The maximum H_2_ concentrations for experiments E1, E2, and E3 were measured to be 0.41%, 0.44%, and 0.86%, respectively. Hence, a higher-temperature environment is essential for achieving a greater degree of gasification of heavy oil and a higher concentration of hydrogen-rich syngas. Furthermore, the total gas production, H_2_-rich gas production, and hydrogen conversion rate are also presented in Figure 5. For experiments E1, E2, and E3, which were conducted without a catalyst, the total gas production increased from 0.028 mol to 0.202 mol and the conversion rate improved from 8.82 mL/g to 134.13 mL/g, while the temperature rose from 400 °C to 600 °C. This improvement was attributed to enhanced cracking efficiency, which implies faster scission reactions for C–C, C–H, and H–H bonds. Subsequently, a greater amount of lighter hydrocarbon compounds was produced as the ambient temperature increased. Scission reactions involving these three types of bonds result in the generation of hydrogen radicals, saturated hydrocarbon radicals, and unsaturated hydrocarbon radicals (both aromatic and non-aromatic) [26]. Some of these free radicals are more likely to engage in cross-bonding reactions to form new molecules, such as the reactions of H· + ·H → H_2_ and H· + ·CH_3_ → CH_4_ [27,28]. Considering the upper temperature limit of the device and the hydrogen production efficiency, 600 °C was selected as the modified ambient temperature of gasification for subsequent experiments. Furthermore, a higher concentration of hydrogen-rich syngas, combined with greater total gas production and higher hydrogen production efficiency, was achieved in experiments E4, E5, and E6 compared to the catalyst-free experiments E1, E2, and E3, as the nanoscale nickel catalyst was used. Notable differences in the CH_4_ concentrations between experiments E3 and E6, including different peak values of 3.96% and 6.13%, were observed despite the experiments being conducted at the same temperature of 600 °C. Such a difference might be attributed to the addition of the catalyst. The maximum H_2_ concentrations in experiments E1–E3 were determined to be 0.41%, 0.44%, and 0.86%, respectively. However, the H_2_ concentrations in experiments E4–E6 significantly increased, with peak values of 0.80%, 1.42%, and 1.74%, due to the effect of the catalyst. Meanwhile, the hydrogen conversion rates of heavy oil in experiments E1–E3 were calculated to be 8.82 mL/g, 33.41 mL/g, and 134.13 mL/g, respectively, and they increased to 13.27 mL/g, 123.40 mL/g, and 228.54 mL/g, respectively, in experiments E4–E6 with the involvement of the catalyst. This improved performance implies that the nickel catalyst has excellent catalytic activity that is characterized by a reduction in the activation energy during the heavy oil gasification process [29,30]. In addition, the catalytic effect can also promote coke deposition, which generates more coke and hydrogen during the gasification process (coke + H_2_O → CO + H_2_) [31].

### 2.3. Analysis of Reaction Kinetics and Ea Fingerprints

**Figure 4:**
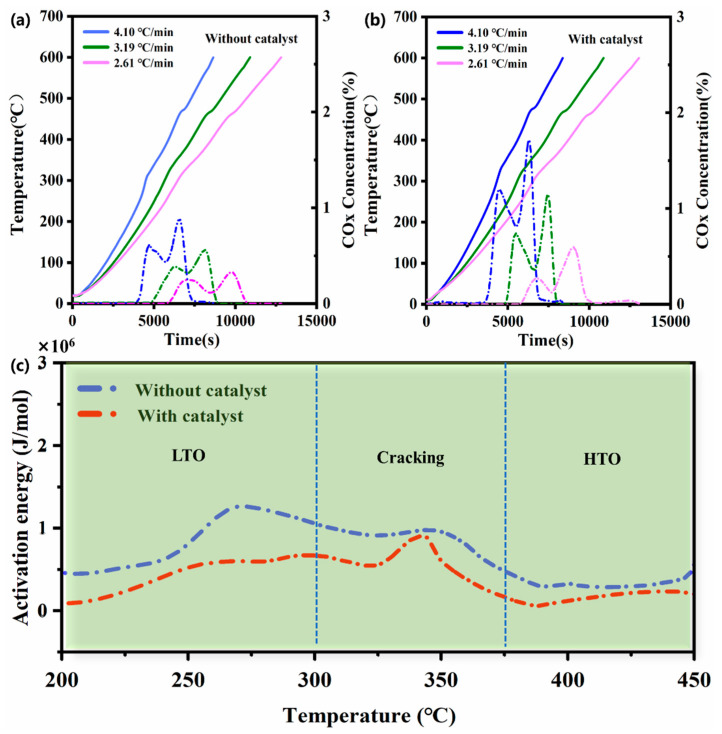
(**a**) Curves of COx concentration (dashed) and temperature (solid) versus time for samples without catalyst. (**b**) Curves of COx concentration (dashed) and temperature (solid) versus time for samples with catalyst. (**c**) Ea fingerprints for samples with and without catalyst.

The variation in the temperature and carbon oxide (CO + CO_2_) concentration over time for two samples, sample A (without the catalyst) and sample B (with the catalyst), is plotted separately in Figure 4a,b. There are two peaks in each temperature curve corresponding to the processes of LTO and HTO. The temperature humps with greater peak values are observed in both low- and high-temperature regions for sample B, which indicates a greater amount of heat generation during the LTO and HTO processes. Hence, the effects of the nanoscale catalyst can be inferred from the significant increase in the reaction rate, and the catalyst can also shift the two oxidation reaction processes to a lower ambient temperature. Taking the heating rate of 4.10 °C/min as an example, the occurrence times of the two temperature peaks of sample A are 78.33 min (299.8 °C) and 113.82 min (450.0 °C), respectively, while for sample B with the catalyst, they decrease to 73.15 min (320.0 °C) and 104.92 min (473.0 °C).

The fingerprints of the Ea versus temperature for the two samples are shown in Figure 4c, which can be separated into three stages. The LTO stage varied within the temperature range of 200–300 °C and was characterized as an oxygen addition process. Then, the cracking stage proceeded when the temperature varied in the range of 300–375 °C. The HTO process occurred in the temperature range of 375–450 °C, and this stage was characterized by the highest amounts of COx generation and heat release. The Ea values of sample B were lower than those of sample A during the LTO, cracking, and HTO stages. Adding the catalyst substantially reduced the energy barrier for the oxidation and cracking reactions of heavy oil, which allowed these reactions to occur in lower-temperature regions and significantly improved the gasification efficiency of the heavy oil. Specifically, the total COx production of sample B was 0.00273 mol at a heating rate of 4.10 °C/min, whereas it decreased to 0.00096 mol for sample A, showing a 64.84% reduction without the catalyst. In addition, the Ea fingerprint of sample A remained relatively stable in the sub-region of 300–375 °C, while for sample B, it had a lower average value accompanied by a greater degree of oscillation. During the cracking process, sample B with the catalyst generated more light components and had a stronger evaporation effect, which led to greater heat loss and a higher degree of oscillation in the Ea fingerprint [32,33].

### 2.4. The Reaction Selectivity of the Catalyst

**Figure 5:**
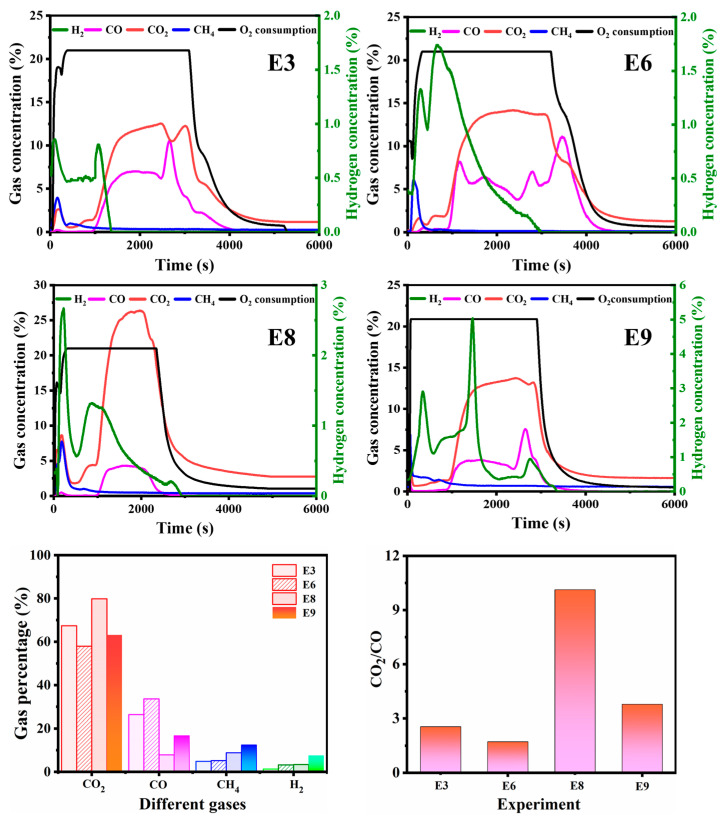
The composition variation of the effluent gases over time in the experiments.

Considering the effect of water in the production of hydrogen-rich syngas, the reaction selectivity of the nickel catalyst was investigated, as shown in Figure 5. The effluent gas concentrations in experiment E3 indicate that hydrogen generation occurred in the period of 0–1700 s with a peak value of around 0.86%. For experiment E6 with the nickel catalyst, however, the peak value of the hydrogen concentration was 1.80%, and the time span of hydrogen generation was extended from 1700 s to 3000 s. When water was presented as a reaction component, such as in experiment E7, the peak value significantly increased to 2.80%. In experiment E8, in the presence of water and the nickel catalyst, the highest peak value of 5.00% (generated at 1750 s) was obtained, and the duration of hydrogen generation was extended to 4000 s. Hence, both the highest peak value and the widest time span of hydrogen generation were obtained with the addition of water and the catalyst. The effect of the water and the catalyst on hydrogen production behavior was also evaluated. First, the reaction selectivity was calculated based on the molar ratio of each produced gas to the total gas production. Then, CO_2_/CO ratios were calculated according to Formula (11). The calculated CO_2_/CO ratios for experiments E3, E6, E7, and E8 were 2.56, 1.73, 10.13, and 3.78, respectively. The CO_2_/CO ratio of E7 was almost 4.0 times that of E3. The significant improvement in hydrogen production in the presence of water was mostly attributable to the water–gas shift reaction (CO + H_2_O → H_2_ + CO_2_). Furthermore, the CO_2_ selectivity in experiments E3 and E7 were 67.42% and 79.85%, respectively. However, as for experiments E6 and E8, the CO_2_ selectivity was reduced to 57.95% and 63.22%, respectively, due to the influence of the catalyst. The variation in reaction selectivity is largely attributed to the effect of the nickel catalyst, which indicates improvements in both reaction activity and selectivity [34,35,36]. The possible reaction schemes are listed as follows:

- Water–gas shift: CO + H_2_O → H_2_ + CO_2_;
- Coke gasification: coke + H_2_O → CO + H_2_;
- Methane steam reforming: CH_4_ + H_2_O → CO + H_2_;
- Methane dry reforming: CH_4_ + CO_2_ → 2CO + 2H_2_;
- Aquathermolysis reaction: C_x_H_y_ + H_2_O → C_m_H_n_ + C+ CH_4_ + CO_2_+ CO + H_2_.

The various reaction pathways in the production of H_2_ and other gases are illustrated in these reactions. Therefore, the effect of the nickel catalyst significantly increases gas production, especially the production of H_2_, CO, and CH_4_.

**Figure 6:**
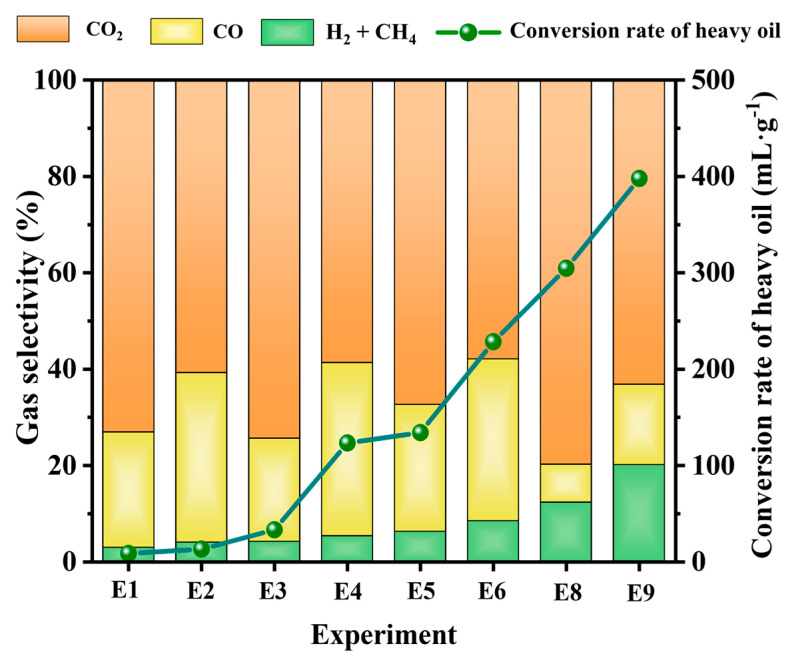
Comparison of gas generation selectivity and hydrogen conversion rates in different experiments.

The reaction selectivity of the gas product yield and the hydrogen conversion rate are presented in Figure 6. The selectivity of hydrogen-rich syngas generation and the hydrogen conversion rate were improved by using the nickel catalyst compared with the catalyst-free experiments. Specifically, the selectivity values of the hydrogen-rich syngas product yields of experiments E1, E2, E3, and E7 were calculated to be 2.91%, 4.17%, 6.22%, and 12.28%, respectively. However, substantial increases in selectivity were obtained, with respective values of 3.99%, 5.28%, 8.44%, and 20.07%, in experiments E4, E5, E6, and E8 upon the addition of the nickel catalyst. Meanwhile, the highest hydrogen conversion rate, with a value of 397.87 mL/g, was achieved in experiment E8. A significant increase in the product yield selectivity of CO, CH_4_, and H_2_, accompanied by a reduction in CO_2_, was observed in all experiments involving the nickel catalyst. For instance, the selectivity values of CO_2_, CO, CH_4_, and H_2_ for experiment E7 were 79.85%, 7.88%, 8.86%, and 3.40%, respectively, while for E8 with the nickel catalyst, these values changed to 63.22%, 16.72%, 12.45%, and 7.62%, respectively. Hence, the prepared nickel catalyst is expected to be a candidate for use in hydrogen production through in-situ gasification of heavy oil, which could achieve high hydrogen-rich syngas production without greatly increasing CO_2_ emissions concerns. Furthermore, the peak hydrogen concentration and hydrogen conversion rate obtained using the nanoscale nickel catalyst in this study were compared with those reported in the literature, as shown in Table 1. The results show that the prepared nanoscale nickel catalyst was proved to be a better catalyst with abundant active sites.

### 2.5. Catalytic Mechanisms for Hydrogen Production

**Figure 7:**
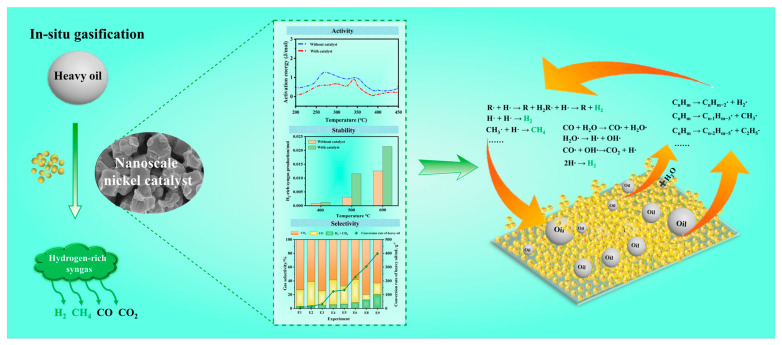
Catalytic mechanism of nanoscale nickel catalyst in hydrogen-rich production during heavy oil gasification process.

The catalytic mechanism of the nanoscale nickel catalyst for hydrogen-rich syngas production is illustrated in Figure 7. In the initial stage of gasification, hydrocarbon compounds of heavy oil are first adsorbed on the surface of the catalyst. The high specific surface area and abundant active sites further promote the adsorption process. Then, the high-molecular-weight hydrocarbons undergo thermal cracking in the high-temperature region to generate hydrocarbon radicals (such as methyl free radicals and ethyl free radicals) [41]. In this process, the nickel catalyst could facilitate the breaking of C–C and C–H bonds of hydrocarbons to form free radicals. Finally, the generated radicals migrate on the surface of the nickel catalyst, where the active sites on the catalyst surface promote radical recombination and hydrogen transfer reactions [42,43]. Then, light hydrocarbon molecules (C_2_H_4_, C_2_H_6_, C_3_H_8_, etc.) and gases (CO, CO_2_, CH_4_, and H_2_) are produced. Furthermore, in the presence of water, CO and H_2_O are also adsorbed and dissociated on the surface of the catalyst, and carbon monoxide radicals (CO·), hydroxyl radicals (·OH), and hydrogen radicals (H·) could be generated. Subsequently, CO· reacts with ·OH to form CO_2_ and H·, and H· combine with each other to form H_2_ [44]. Coke deposition refers to the dehydrogenation process of heavy components on the catalyst surface, which hinders catalytic activity by isolating the active sites. Coke would be consumed as a fuel at an even higher ambient temperature to sustain the HTO reactions. Additionally, coke can also react with H_2_O and CO_2_ in the high-temperature region (C + H_2_O → CO + H_2_, C + CO_2_ → 2CO) to generate syngas (H_2_ and CO), which greatly reduces coke deposition and improves gasification efficiency. Consequently, the catalyst deactivation caused by the carbon deposition process can be effectively reduced when the nanoscale nickel catalyst is presented during the heavy oil gasification process.

## 3. Experiments

### 3.1. Materials

The heavy oil sample used in this study was obtained from the CG* block of the Shengli oil field in Shandong province, China. The dewatering process was performed using an electric dehydration instrument (Petroleum Analytical Instrument Company, China). Then, the viscosity of the oil sample was measured using an HAAKE RS6000 Rotor Rheometer (Thermo Scientific, Germany). The measured value was 73.4 Pa·s at a shear rate of 10 s⁻^1^ and a temperature of 30 °C. The density of the oil was 0.98 g/cm^3^ in standard ambient conditions. Quartz sand with a particle size of 40–60 mesh was used in a kinetic cell experiment. Nickel chloride (NiCl_2_, 99%) and the reducing agent sodium borohydride (NaBH_4_, 95%) were purchased from Xinbaohai Chemical Technology Co., Ltd., Beijing, China. The anionic surfactant sodium dodecyl benzene sulfonate, the co-surfactant n-hexanol, cyclohexane, and a conventional nickel catalyst (0.5–1.5 μm) were purchased from Kemiou Chemical Reagent Co., Ltd., Tianjin, China. Analytical-grade methanol and ethanol were purchased from Tianjin Tianli Chemical Reagent Co., Ltd., Tianjin, China. Distilled water was used for the preparation of all aqueous solutions.

### 3.2. Preparation and Characterization of the Catalyst

**Figure 8:**
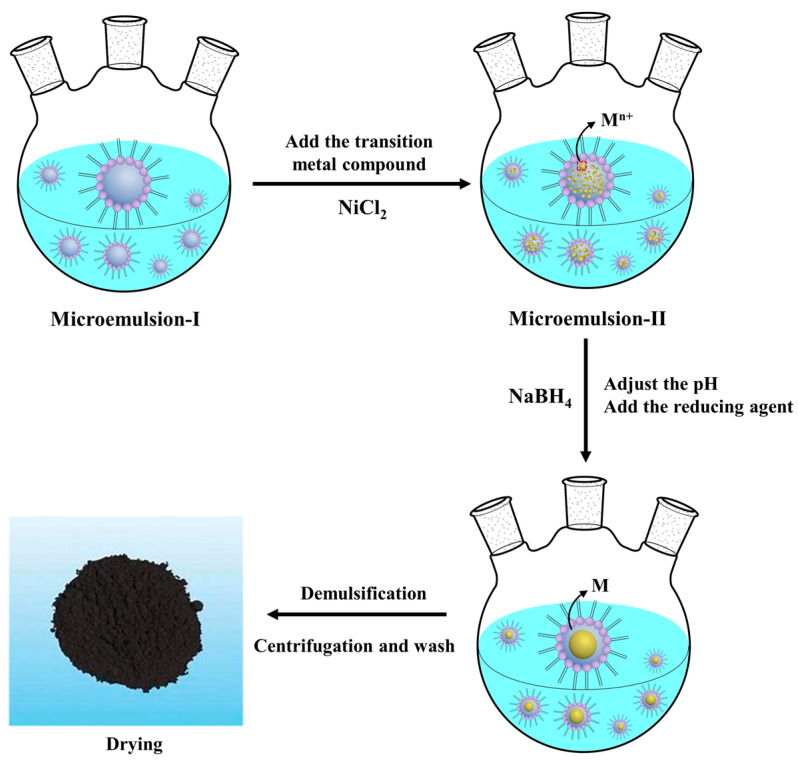
A schematic of the preparation of the nanoscale nickel catalyst.

The preparation of the nanoscale nickel catalyst is illustrated in Figure 8. First, the oil phase (cyclohexane), water, surfactant (SDBS), and co-surfactant (n-Hexanol) were mixed at a volume ratio of 50:10:1:0.2 to form a multiphase dispersion system. Then, they were stirred for 2 h until a homogeneous, thermodynamically stable, and transparent water-in-oil (W/O) microemulsion was obtained (microemulsion-I). Second, 3 mL of a NiCl_2_ solution (3.33 mol/L) was slowly added to microemulsion-I and stirred for 1 h to yield a uniform colloid system (microemulsion-II). Third, the pH of microemulsion-II was adjusted to 11 ± 0.5 using a sodium hydroxide solution with a concentration of 0.5 mol/L. Then, 4 mL of a NaBH_4_ (2 mol/L) solution was added drop-wise to the microemulsion-II system while the ambient temperature was maintained using an ice bath. The solution was stirred vigorously to ensure that local overheating and boiling were prevented. The system was allowed to settle until the color changed to grayish black. Finally, an excess of methanol was added for demulsification. Then, the generated nickel nanoparticles were collected through a centrifugation process and washed sequentially with water and ethanol three times to remove organic residue. Subsequently, they were dried at 110 °C for 10 h. The morphology and size of the nanoparticles were characterized using scanning electron microscopy (SEM, Carl Zeiss, Germany). The surface properties of the nano-nickel were determined by a N_2_ adsorption/desorption tester (Autosorb IQ2, Quanta chrome, Boynton Beach, FL, USA) at 196 °C.

### 3.3. Catalytic Tests

**Figure 9:**
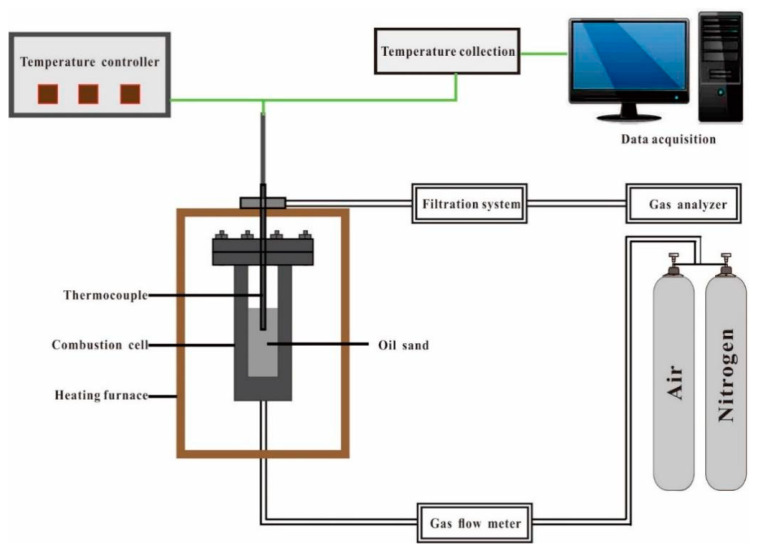
A schematic diagram of the kinetic cell experiment.

The catalytic effect of the nanoscale nickel catalyst on hydrogen-rich gas generation during in-situ gasification of heavy oil was evaluated using kinetic cell experiments. A schematic diagram of the apparatus is presented in Figure 9. The device contained four experimental units, including a heating and temperature control unit, a gas flow rate control unit, a gas concentration measurement unit, and a data logging and transportation unit. The quartz sand particles were adequately blended with heavy oil at a mass ratio of 15:2.5. To minimize the mass loss caused by the drainage and vaporization of the oil, 15.5 g of the oil–sand mixture was precisely weighed and placed in the cell as the middle layer. This middle layer was paved with a layer of clean quartz sand weighing 5.0 g as the bottom layer and covered with 7.0 g of identical sand as the top layer. Upon the introduction of the catalyst, it was blended with the heavy oil with a constant catalyst-to-oil mass ratio of 0.2:1. More information on the procedures is presented in our previous research [45]. The parameters of the kinetic cell experiments are listed in Table 2.

The effluent gas concentrations (CO, CO_2_, O_2_, CH_4_, and H_2_) were monitored in real time using a gas analyzer from Wuhan Cubic Optoelectronics Co., Ltd., Wuhan, China. The concentration data were recorded automatically each second by the data logger installed in the computer. The mole amount of gas production, the total gas production, the hydrogen-rich syngas production, and the gas selectivity were calculated using the equations below. The hydrogen conversion rate of heavy oil (C*_hydrogen–oil_*) is defined as the ratio of the total volume of hydrogen-rich syngas generated (n*_hydrogen-rich syngas_*) to the initial weight of the heavy oil (m_oil_). It reflects the volume of hydrogen-rich syngas that can be generated per gram of heavy oil.

(1) $$n_{H_{2}}=q\cdot \int_{0}^{t}\left(C_{H_{2}}\right)dt/22.4$$

(2) $$n_{CH_{4}}=q\cdot \int_{0}^{t}\left(C_{CH_{4}}\right)dt/22.4$$

(3) $$n_{CO}=q\cdot \int_{0}^{t}\left(C_{CO}\right)dt/22.4$$

(4) $$n_{CO_{2}}=q\cdot \int_{0}^{t}\left(C_{CO_{2}}\right)dt/22.4$$

(5) $$n_{total}=q\cdot \int_{0}^{t}\left(C_{H_{2}+CH_{4}+CO+CO_{2}}\right)dt/22.4$$

(6) $$n_{hydrogen-rich\cdot syngas}=q\cdot \int_{0}^{t}\left(C_{H_{2}+CH_{4}}\right)dt/22.4$$

(7) $$S_{H_{2}}=\frac{n_{H_{2}}}{n_{total}}\times 100\%$$

(8) $$S_{CH_{4}}=\frac{n_{CH_{4}}}{n_{total}}\times 100\%$$

(9) $$S_{CO}=\frac{n_{CO}}{n_{total}}\times 100\%$$

(10) $$S_{CO_{2}}=\frac{n_{CO_{2}}}{n_{total}}\times 100\%$$

(11) $${\;\mathrm{CO}}_{2}/\mathrm{CO}=\frac{S_{{CO}_{2}}}{S_{CO}}$$

(12) $$C_{hydrogen-oil}=\frac{n_{hydrogen-rich\cdot syngas}}{m_{oil}}$$

where q represents the gas flow rate (L/min); t is the duration of different reaction processes (s); C_x_ is the concentration of the produced gases (H_2_, CH_4_, CO, and CO_2_) (%); S_x_ represents the selectivity for the produced gases (H_2_, CH_4_, CO, and CO_2_); C_hydrogen–oil_ is the hydrogen conversion rate of the heavy oil (mL/g); and m_oil_ represents the initial weight of the heavy oil (g).

### 3.4. Reaction Kinetics

Kinetic cell experiments with ramped temperature oxidation (RTO) were conducted to obtain apparent activation energy (Ea) fingerprints during reactions of heavy oil and oxygen with and without the nanoscale nickel catalyst. The sample preparation processes were consistent with those described in Section 2.3. Prescribed constant heating rates (2.61 °C/min, 3.19 °C/min, and 4.10 °C/min) were programmed into the furnace controller, with starting and setting temperatures of 25 °C and 600 °C, respectively. The heating process was terminated when the temperature reached the target of 600 °C. The parameters of the RTO experiments are listed in Table 3.

Temperature and COx concentration data for three different heating rates were processed using the iso-conversional method [46]. Then, the Ea was calculated using the Friedman method [47]. This detailed calculation was initially described by Cinar et al. and was further elaborated in our previous work [48,49]. With the iso-conversional method and the Arrhenius equation, the reaction kinetic equation is expressed using Equations (13) and (14).

(13) $$\frac{d\alpha }{dt}=f\left(\alpha \right)k\left(T\right)$$

(14) $$\ln\left(\frac{d\alpha }{dt}\right)=\ln\left[Af\left(\alpha \right)\right]-\frac{E_{\alpha }}{RT}$$

where *α* is conversion; *t* is time in seconds; *f*(*α*) is the reaction mechanism function, which depends only on the degree of conversion; *k*(*T*) is the reaction rate constant, which depends only on the temperature; A is the frequency factor; Ea is the activation energy in KJ/mol; R is the ideal gas constant, 8.314 J/(mol·K); and *T* is the absolute temperature in K.

## 4. Conclusions

In this work, a nanoscale nickel catalyst was prepared, and its application in hydrogen-rich syngas generation through the in-situ combustion gasification of heavy oil was investigated. Preliminary results were obtained, and further investigation of the mechanism is needed in the future. The conclusions are summarized as follows:

(1) The nanoscale nickel catalyst was synthesized by the reverse microemulsion method, and it exhibited a cubic morphology with a size distribution ranging from 50 to 90 nanometers.

(2) The mechanisms of hydrogen-rich syngas production with the nanoscale nickel catalyst were mainly manifested in six aspects: reductions in the Ea value, the CO_2_/CO ratio, and the temperature thresholds of gasification and increases in the peak value of the hydrogen concentration, total gas production, and the hydrogen-rich syngas conversion rate of heavy oil.

(3) The nanoscale nickel catalyst exhibited excellent catalytic activity and high selectivity in hydrogen-rich syngas production during the in-situ combustion gasification process. The hydrogen concentration reached a maximum value of 5%, coupled with a H_2_ selectivity value of 20.07% and a hydrogen-rich syngas conversion rate of 397.87 mL/g.

## Figures and Tables

**Table 1:** A comparison of catalytic performance between the prepared catalyst and others reported in the literature.

Catalyst	Hydrogen Conversion Rate of Heavy Oil (mL/g)	Peak Hydrogen Concentration (%)	Reference
Iron-activated carbon	157.63	−	Yuan et al. [37]
Iron–silicon carbide	231.81	−	
Iron catalyst	280	−	Yan et al. [38]
Nickel–alumina catalysts	310	−	Guus et al. [39]
Nickel nitride catalyst	−	1.50	Yi et al. [12]
Rock mineral (10–20 mesh)	−	2.24	Jin et al. [40]
Rock mineral (40–60 mesh)	−	2.32	
Rock mineral (>100 mesh)	−	2.35	
Core	−	5.01	
Nano-nickel catalyst	397.87	5.00	This work

**Table 2:** The evaluation of hydrogen-rich syngas generation through heavy oil conversion under different reaction conditions.

No.	Materials Used	Heavy Oil, g	Catalyst, g	Water, mL	Temperature, °C
E1	Heavy oil + quartz sand	2.5	0	0	400
E2	Heavy oil + quartz sand + nanoscale nickel catalyst	2.5	0.25	0	400
E3	Heavy oil + quartz sand	2.5	0	0	500
E4	Heavy oil + quartz sand + nanoscale nickel catalyst	2.5	0.25	0	500
E5	Heavy oil + quartz sand	2.5	0	0	600
E6	Heavy oil + quartz sand + nanoscale nickel catalyst	2.5	0.25	0	600
E7	Heavy oil + quartz sand + conventional nickel catalyst	2.5	0.25	0	600
E8	Heavy oil + quartz sand + water	2.5	0	4	600
E9	Heavy oil + quartz sand + water + nickel catalyst	2.5	0.25	4	600

**Table 3:** Experimental conditions for determining activation energy of heavy oil at different heating rates.

No.	Sand Pack	Heating Rate, °C/min	Temperature, °C	Pressure, MPa
E10	Oil sand	2.61	25–600	1
E11	Oil sand	3.19	25–600	1
E12	Oil sand	4.10	25–600	1
E13	Oil sand + catalyst	2.61	25–600	1
E14	Oil sand + catalyst	3.19	25–600	1
E15	Oil sand + catalyst	4.10	25–600	1

## Data Availability

The original contributions presented in this study are included in the article. Further inquiries can be directed to the corresponding author.
